# Uncommon Presentation of Isolated Jejunal Lymphoma Masquerading as Crohn's Disease

**DOI:** 10.1155/2016/5603627

**Published:** 2016-05-23

**Authors:** Swati Sattavan, Lalit Aggarwal, Priyadarshi Dikshit

**Affiliations:** ^1^Dr. Ram Manohar Lohia Hospital and PGIMER, New Delhi 110001, India; ^2^Lady Hardinge Medical College and Associated Hospitals, New Delhi 110001, India

## Abstract

Primary gastrointestinal lymphoma is a rare entity, commonly involving stomach, small bowel, and colorectum. The usual location for small bowel B cell lymphoma is distal ileum due to abundant lymphoid tissue. We are reporting the case of a 53-year-old lady presumptively diagnosed as Crohn's disease on clinical and radiological grounds but histopathologically proven to be an unusual variant of isolated primary non-Hodgkin's lymphoma.

## 1. Introduction

Gastrointestinal (GI) lymphomas may clinically be confused with other GI tumours as well as with inflammatory bowel disease. About 5% of all lymphomas primarily involve the GI tract. The small intestine is the site of origin of about 15–25% of primary GI lymphomas, with stomach being the most common site affected. GI lymphomas arise from the lymphoid tissue within the wall of small intestine which is abundant in the ileum. Primary GI lymphoma is usually localized to a single segment of the intestine, with multicentricity seen in 15–20% of cases [1]. The pathogenesis of GI lymphomas is still unclear; however, immunogenic alterations have been suspected to play a role.

We report the rare case of a 53-year-old lady presenting with features, which was clinic-radiologically diagnosed to be jejunal stricture secondary to Crohn's disease (CD) on the basis of clinical and radiological features and was reported to be a case of non-Hodgkin's lymphoma (NHL) of B cell type (uncommon in jejunum) on histological evaluation. Our report emphasizes the importance of considering lymphoma in the differential diagnosis of jejunal stricture presenting with small bowel obstruction.

## 2. Case Report

A 53-year-old lady presented to us with chief complaint of left-sided abdominal pain associated with multiple episodes of bilious vomiting over duration of 3 months. She had episodes of diarrhoea for the initial week of onset of symptoms which had improved with oral antibiotics. There was no history indicative of immunosuppression. The general physical and abdominal examinations of the patient were within normal limits. Her blood parameters were within normal limits. Ultrasonography of abdomen revealed evidence of concentric wall thickening of small bowel loops in the left lumbar and paraumbilical region measuring approximately 9 mm with loss of wall stratification. Contrast Enhanced Computed Tomography (CECT) abdomen showed stricture with thick enhancing walls in proximal jejunum measuring 4 cm in length with surrounding fat stranding and dilated proximal and collapsed distal bowel loops. A focal area of proximal jejunum was also thickened. It also showed evidence of hyperemia with dilated mesenteric vessels. These features were suggestive of inflammatory pathology, with a differential diagnosis of Crohn's disease or chronic infectious process such as tuberculosis. Colonoscopy was unremarkable.

**Figure 1:**
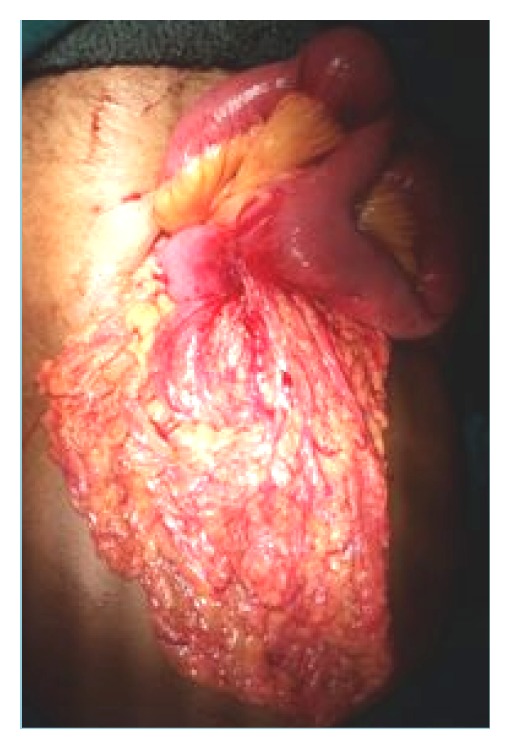
The intraoperative findings showing the jejunal loop and the adjacent mesentery and the segment of transverse colon.

**Figure 2:**
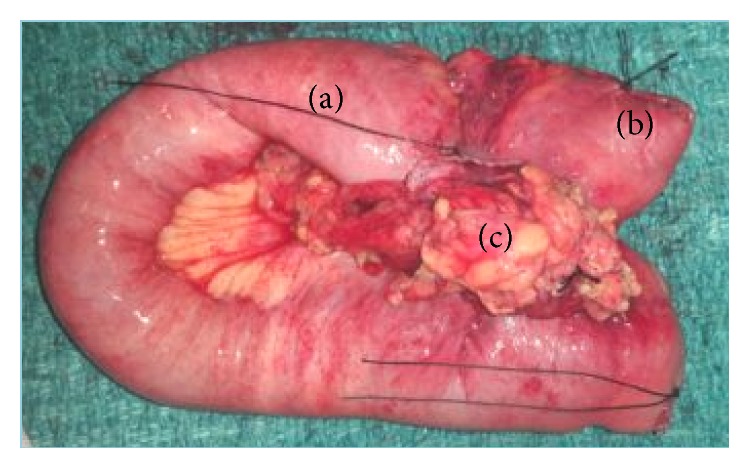
The resected specimen showing the affected jejunal segment (a) and adherent mesentery (c) with resected segment of transverse colon (b).

She was thereafter planned for surgery. Intraoperatively there was a jejunal stricture present at approximately 1.5 feet distal to duodenojejunal flexure with surrounding fibrosis and mesentery and transverse colon adhered to the segment with associated mesenteric lymphadenopathy (Figures 1 and 2).

The histopathology report showed diagnosis of non-Hodgkin's lymphoma of B cell type. Immunohistochemistry was positive for CD20, CD3, and LCA and was negative for CD117 and Pan CK. The postoperative period of the patient was uneventful and the patient was given the chemotherapy based on R-CHOP regimen (rituximab, cyclophosphamide, doxorubicin, vincristine, and prednisone).

## 3. Discussion

Primary gastrointestinal non-Hodgkin's lymphomas (PGINHL) are one of the rare lymphoproliferative neoplasms arising from lymphatic tissue below the mucosal membrane of the gastrointestinal tract (GIT), accounting for less than 5% of GIT lymphomas [2]. The criteria (Dawson et al.'s) to define primary gastrointestinal (GI) lymphoma are as follows: (a) the GI tract is predominantly affected with lymph node involvement confined to drainage area of the primary site; (b) there is no hepatic or splenic involvement or palpable superficial node; (c) normal chest radiograph; and (d) the peripheral white blood cell count is normal [3]. The association between CD and NHL is still controversial, regarding whether it is due to disease activity or an effect of the medical therapy. NHL occurring in patients with CD is usually extraintestinal. Lymphoma is the most common malignancy of small bowel, mostly located in the distal ileum due to presence of lymphoid tissue in case of B cell type. On the other hand, T cell lymphoma is more common in jejunum [4]. B cell type tends to form annular to polypoid masses in distal or terminal ileum, whereas T cell type appears as ulcerated plaque or stricture in the proximal small intestine. It is very unlikely for lymphoma to present as bowel obstruction due to lack of desmoplastic reaction. In our case, however, presentation was in the form of recurrent obstruction secondary to a stricture in the proximal jejunum which was due to lymphoma of B cell type. The episodes of diarrhoea in the initial period might have been secondary to superimposed gastroenteritis as the patient belonged to low socioeconomic status and it responded to the antibiotic treatment.

PGINHL is usually associated with haemorrhage and perforation and rarely with stricture formation. Although stricture formation might be one of the predictable complications of PGINHL, it has not been reported in previous studies; in a prospective trial of 56 patients with intestinal lymphoma, there were no reported cases of bowel stricture [5]. Up to one-half of patients with intestinal lymphoma present with acute abdomen. Imaging studies may include CECT of abdomen and pelvis, lymphangiography, radionucleotide scanning, upper GI contrast studies, barium enema, esophagogastroduodenoscopy, or colonoscopy, depending on the lesion location and clinical presentation.

The main modality of treatment for intestinal lymphoma is chemotherapy. Surgical intervention is usually reserved for complicated lymphomas like in obstruction or perforation. The most common chemotherapeutic regimen includes R-CHOP for B cell lymphoma.

## 4. Conclusion

Intestinal lymphomas may masquerade as Crohn's disease or chronic infectious disorders. On the other hand, there may be some causal association between the two pathologies, although so far it is unclear. Hence a differential diagnosis of lymphoma should always be considered while dealing with such patients. Endoscopy with deep biopsies followed by immunophenotypic studies should be considered as a part of evaluation so as to reach to a definite diagnosis and timely intervention.
